# Intermediate Coupling between Aboveground and Belowground Biomass Maximises the Persistence of Grasslands

**DOI:** 10.1371/journal.pone.0061149

**Published:** 2013-04-29

**Authors:** Simon Scheiter, Steven I. Higgins

**Affiliations:** 1 Biodiversität und Klima Forschungszentrum (LOEWE BiK-F), Senckenberg Gesellschaft für Naturforschung, Frankfurt am Main, Germany; 2 Institut für Physische Geographie, Johann Wolfgang Goethe-Universität Frankfurt am Main, Frankfurt am Main, Germany; University of Zurich, Switzerland

## Abstract

Aboveground and belowground biomass compartments of vegetation fulfil different functions and they are coupled by complex interactions. These compartments exchange water, carbon and nutrients and the belowground biomass compartment has the capacity to buffer vegetation dynamics when aboveground biomass is removed by disturbances such as herbivory or fire. However, despite their importance, root-shoot interactions are often ignored in more heuristic vegetation models. Here, we present a simple two-compartment grassland model that couples aboveground and belowground biomass. In this model, the growth of belowground biomass is influenced by aboveground biomass and the growth of aboveground biomass is influenced by belowground biomass. We used the model to explore how the dynamics of a grassland ecosystem are influenced by fire and grazing. We show that the grassland system is most persistent at intermediate levels of aboveground-belowground coupling. In this situation, the system can sustain more extreme fire or grazing regimes than in the case of strong coupling. In contrast, the productivity of the system is maximised at high levels of coupling. Our analysis suggests that the yield of a grassland ecosystem is maximised when coupling is strong, however, the intensity of disturbance that can be sustained increases dramatically when coupling is intermediate. Hence, the model predicts that intermediate coupling should be selected for as it maximises the chances of persistence in disturbance driven ecosystems.

## Introduction

Aboveground and belowground biomass compartments of vegetation are coupled by fluxes of water, nutrients and carbohydrates and this coupling strongly influences the dynamics and persistence of ecosystems. One aspect of coupling is that roots and shoots fulfil different functions and thereby co-limit plant growth. Aboveground biomass is responsible for carbon uptake by photosynthesis while belowground biomass is responsible for nutrient and water uptake from the soil. Subsequent to their uptake, carbon, nutrients and water are exchanged between aboveground and belowground biomass compartments of the plant. Such exchanges are well supported empirically, for example it has been observed that more than 60% of the carbon fixed by photosynthesis can be allocated to roots [1] and that approximately 75% of the nitrogen acquired by roots can be allocated to shoots [2]. The coupling of aboveground and belowground biomass additionally allows plants to recover from injuries caused by disturbance, such that vegetation dynamics are buffered to disturbances. For instance, in ecosystems such as tropical grasslands and savannas, aboveground biomass is regularly reduced by fire and herbivory [3]. After such disturbances, belowground storage resources allow plants to resprout and to produce new shoots that in turn can assimilate carbon [4], [5].

Despite the evidence that coupling of aboveground and belowground biomass occurs and despite our knowledge of the importance of coupling for ecosystem resilience, existing heuristic ecological models often use a single compartment structure. These models typically use only one state equation to describe the dynamics of both aboveground and belowground biomass (e.g. the logistic equation or Volterra-Lotka-type coexistence models [6]). Such single compartment models are attractive due to their simple equations which often allow mathematical analyses. Further, single compartment models often serve to describe fundamental ecosystem dynamics in economic models. However, they do not provide a description of root-shoot coupling and how this might buffer a system's response to injury.

Although many heuristic models use a single compartment approach, several studies have adopted a multi-compartment approach. For instance, models that separate between leaf, stem and root carbon and nitrogen pools in vegetative plants have been developed to explore how uptake, transport and utilisation of resources respond to photosynthetic and nutrient uptake rates [7]. In several subsequent studies this model approach has been modified to simulate roots and shoots only [8]–[10] or to include physiological processes that allow to project species distribution patterns [11]. Roots, stems and leaves have been separated to explore carbon allocation patterns in a forest system [12]. Models that separate biomass were used to define optimal and sustainable rangeland management strategies [13]–[15]. More specifically, grass biomass was split into shoots and crowns/roots [14] and into green (photosynthetic) and non-structural carbon reserves [15]. Alternative models distinguish between biomass compartments in and out of the flame zone in a forest model and show that growth and flammability of the two biomass compartments influence fire regimes [16]. In a previously presented heuristic savanna model [17], [18] we separated biomass of grasses and trees into aboveground and belowground biomass compartments and linked these compartments by assuming that aboveground and belowground biomass growth is influenced both by aboveground and belowground biomass. In these studies we explored aspects of optimal grazing strategies, fire regimes and coexistence. We did, however, not explicitly explore how the strength of coupling aboveground and belowground biomass compartments influences the system dynamics and the root buffering capacity. We rather assumed that roots and shoots are strongly coupled, that is that growth of roots is exclusively determined by shoots while the growth of roots is exclusively determined by shoots [18]. However, the assumption of strong coupling is an oversimplification of root-shoot dynamics as in reality, growth of a plant's biomass compartment is not solely determined by the other plant compartments, it is rather co-limited by the abundances of both compartments [8].

In this paper, we use a grassland model derived from a heuristic savanna model [17], [18] to explore how the coupling strength of aboveground and belowground biomass compartments influences the system's dynamics. Specifically, we examine the long term maximum aboveground biomass that can be removed by fire and grazing, without driving the system towards a collapse. We show that the maximum biomass removal and ecosystem behaviour are strongly influenced by the strength of coupling of aboveground and belowground biomass compartments and that intermediate levels of coupling optimise the trade-off between productivity and persistence of the system.

## Models

The grassland model we explore is based on a previously presented savanna model [17], [18]. The grassland model distinguishes between an aboveground and a belowground biomass compartment, that is, between shoots 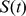 and roots 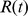
Here, 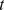 denotes the time. The two state variables are assumed to be abundances between zero and one.

We assume that the growth rates of the two biomass compartments are co-limited by both the root and the shoot biomass [7] and we describe this effect by using the growth function 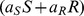. The parameter 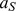 describes the contribution of shoot biomass, which is responsible for photosynthetic carbon gain, to vegetation growth. The parameter 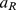 describes the contribution of root biomass, which is responsible for water and nutrient uptake, to vegetation growth. We further assume that after biomass removal by disturbances such as fire or herbivory, vegetation tends to recover and to restore an equilibrium root-shoot ratio [4], [5]. We therefore use the function 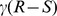 to describe how the biomass compartment with the higher abundance supports regrowth of the biomass compartment with the lower abundance and thereby buffers vegetation dynamics. Here, 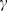 is a constant parameter in the interval 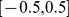. Should for instance shoot biomass 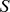 be reduced by fire, then the growth rate of shoots increases by 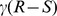 while the growth rate of roots decreases by the same amount. This function implies that the strength of this effect decreases when the difference between root and shoot abundances decreases. Hence, in the model, vegetation behaves to maintain an equilibrium between abundance and growth of shoots and abundance and growth of roots [19]. With these assumptions, we define the growth functions of shoots and roots as
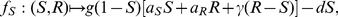
(1)

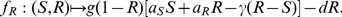
(2)


In eqn (1) and eqn (2), 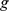 is a constant growth parameter and 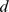 describes the aggregated effect of mortality, respiration and decomposition. Multiplying 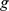 by 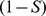 and 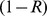 respectively, ensures that growth is density dependent. Should for instance 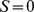 then shoot growth is 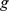 whereas it tends to zero when 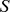 approaches one. For the model to be biologically reasonable, the parameters 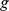 and 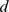 must be greater than zero and the growth parameter 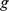 must be greater than the mortality parameter 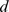, that is, 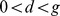. For simplicity, we assume that shoot and root abundances are equally important for plant growth, that is 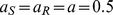. Then, eqn (1) and eqn (2) can be expressed as
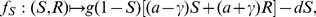
(3)

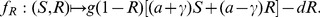
(4)


As 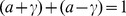 we can define 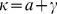 and re-write the growth functions as
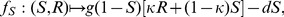
(5)

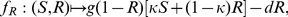
(6)


where a single parameter 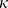, hereafter denoted as coupling parameter, describes how shoots contribute to the growth of roots and how roots contribute to the growth of shoots. When 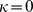, then the dynamics of shoots and roots are decoupled and the compartments do not interact, that is, roots do not influence the growth of shoots and vice versa. This case is biologically implausible and ignored here. When 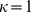, then shoots and roots are fully coupled, which means that the growth of shoots is solely defined by roots and that the growth of roots is solely defined by shoots. This case is also biologically not reasonable, however, it is helpful for the model analysis. We denote 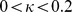 as weak coupling, 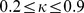 as intermediate coupling, 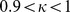 as strong coupling and 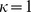 as full coupling.

Considering fire and grazing, the growth functions in eqn (5) and eqn (6) can be written as
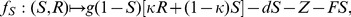
(7)

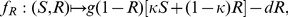
(8)


where 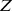 and 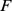 describe shoot biomass loss due to grazing and fire, respectively. We use the grazing function
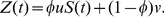
(9)


The grazing model assumes that the offtake of aboveground biomass is a mixture of two offtake processes. In the first process, a fixed fraction 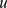 of shoot biomass 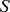 is removed in each time interval. In the second process, a fixed amount of biomass 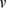 is removed in each time interval. Both 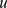 and 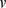 are non-negative constants. The parameter 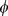, which is between zero and one, defines the mixing ratio of these two processes. The grazing function allows the definition of two simple, but fundamentally different grazing strategies. When 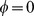, then 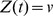 which means that a fixed amount of biomass is removed from the system in every time interval (hereafter called the ″fixed offtake strategy″). Hence, the biomass removal is constant and not adjusted to the available shoot biomass. In contrast, when 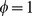, then 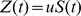 which means that a fixed fraction of the shoot biomass is removed in each time interval (hereafter called ``fixed fraction strategy''). In this fixed fraction strategy, biomass removal by grazing is adjusted to the available shoot biomass.

Of course, both the fixed offtake strategy and the fixed fraction strategy are oversimplifications. In reality, the grazing intensity cannot be perfectly adjusted to the shoot biomass 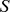, neither by a farmer selling and buying animals nor by a natural reproduction and mortality process of grazers. On the other hand, a constant number of grazers cannot be maintained for such a long time period as we assume here. Grazing in both a farm and in a natural ecosystem would be a mixture of these processes which can be mimicked by choosing an intermediate value for 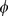. Various studies provide more detailed analyses of alternative strategies in the context of optimal grazing [17], [20]–[23].

We model fire as a discontinuous event that occurs at a fixed fire return interval 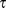. We assume that fire instantaneously consumes the total aboveground biomass 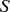 while belowground biomass 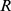 is influenced by fire only indirectly by the removal of shoot biomass. Between two fire events, vegetation grows in absence of any fire impact (eqn. 7 and 8), which allows vegetation to recover. The function 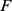 describing fire effects is given by
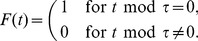
(10)


This function imitates management fires at fixed return intervals while it ignores the fact that natural fire regimes are primarily defined by fuel biomass and fuel moisture [24]. This fire function could also be interpreted as regular harvesting.

The trajectory describing grassland dynamics is given as the solution of the system of differential equations 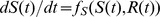 and 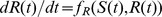, given initial values 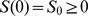 and 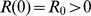 at 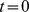.

## Results

### Full coupling

We conduct a fixed-point analysis of the grassland model. We first assume full root-shoot coupling and no grazing, that is 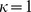 and 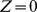. We analyse the case 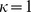 as it has the same fixed points as the cases 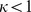 while it simplifies the analyses. Solving the fixed-point equations 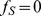 and 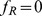 gives the trivial fixed-point
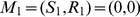
(11)


and a fixed-point
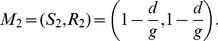
(12)


The Jacobian of the system is given by
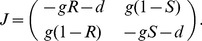
(13)


The eigenvalues of the Jacobian evaluated for the fixed-points 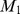 and 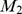 are
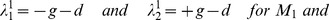
(14)

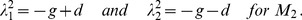
(15)


Due to the assumption that 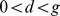, the eigenvalues 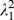 and 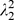 are less then zero such that 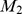 is asymptotically stable. Hence, all trajectories converge towards 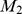 as long as the initial root or shoot abundance is greater than zero, which we assumed.

#### Grazing

The following analysis explores the yield-effort relationship for the model. When shoots and roots are fully coupled (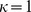) we can calculate an analytic solution for the maximum grazing rates 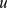 under the fixed fraction strategy (when 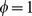) and 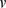 under the fixed offtake strategy (when 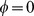) and the maximum grazing yield 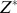 that can be removed from the system. For the case of fixed fraction grazing (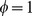), the fixed points are given by 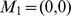 and by
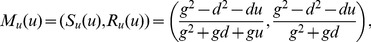
(16)


which is asymptotically stable as long as 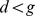 and as long as the grazing rate 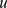 does not exceed the maximum grazing rate 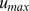, defined by
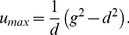
(17)


**Figure 1:**
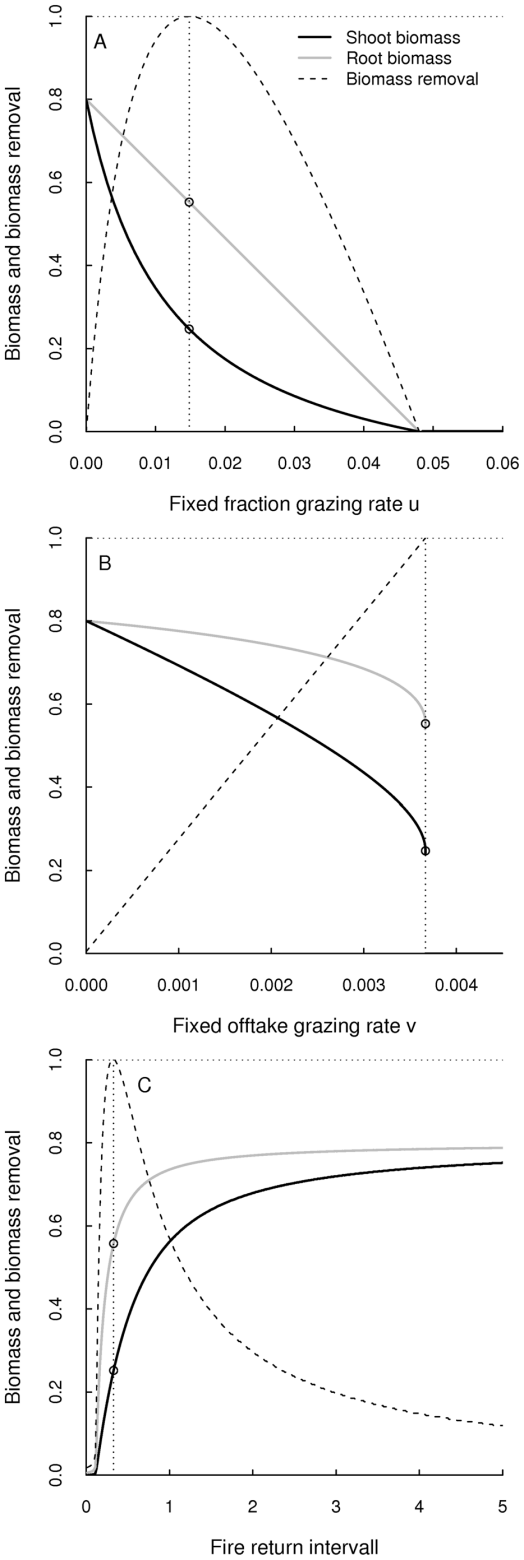
Grazing and fire in fully coupled system. Equilibrium shoot and root biomasses and the biomass removal (as a proportion of 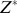) under different grazing and fire strategies and intensities. The panels depict (A) the fixed fraction strategy (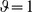) with variable parameter 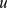, (B) the fixed offtake strategy (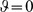) with variable parameter 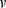 and (C) the fire model with variable return interval 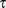. The small circles indicate the equilibrium biomasses 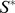 and 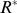 when the maximum biomass 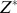 is removed. For these plots we used 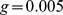 and 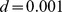.



When the grazing intensity 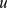 exceeds 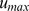, then the shoot biomass loss by grazing is too high to be balanced by roots and the system collapses, that is the system converges to the fixed point 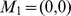 which is then asymptotically stable attractor (Fig. 1A). The maximum grazing yield is given by

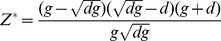
(18)which is obtained when the system is grazed with the optimum grazing rate
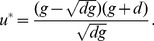
(19)


In this situation, the equilibrium biomass is given as
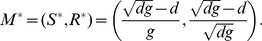
(20)


When the system is grazed with a fixed offtake strategy (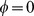), then the fixed points defined by the solutions of 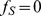 and 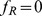 are
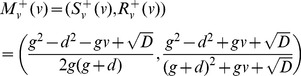
(21)


and
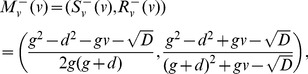
(22)


where
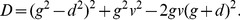
(23)


When the expression 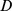 is positive, then real solutions for 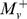 and 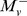 exist. The expression 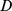 is positive as long as 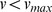 where 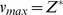 (eqn 18). In such a situation, 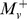 is asymptotically stable while 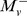 is unstable (Fig. 1B). Hence, when the initial state variables 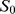 and 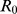 exceed 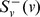 and 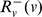 respectively, then the trajectory converges towards 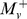 while the system collapses when the initial state variables 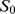 and 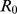 are less than 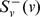 and 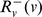. When 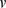 is equal to 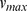 then 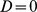 and 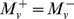. Hence, the system has only one fixed point. This fixed point can be shown to be 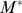 as given by eqn (20). When 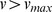 then the root of 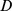 has no real solutions and the system collapses, independent of the initial conditions.

These analyses show that the presented two compartment grassland model provides yield-effort relationships similar to those established for one compartment models such as the logistic equation [6].

#### Fire

We first consider the response of the system to a single fire event. Assume that a fire has reduced shoot biomass to zero. Then from eqn (8) it is clear that in the case of full coupling (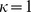) the growth rate of roots is solely determined by decomposition and is therefore negative or zero. Hence, roots react instantaneously to shoot removal by stopping growth and loosing biomass due to decomposition. However, as long as root biomass is greater than zero, shoots have a positive growth rate (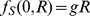) and can therefore recover from disturbance. That is, roots subsidise shoot regrowth to re-establish a balanced root-shoot ratio. After some time, the system will completely recover and reach the stable equilibrium 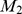 again. The time needed to recover to the equilibrium depends on the root biomass at fire ignition. A higher root biomass implies a higher buffering capacity and faster recovery compared to situations where root biomass is low at fire ignition.

When the system is affected by regular fires, then these fires impose periodicity and event-to-event dynamics [25] on the trajectory: a fire instantaneously reduces the shoot biomass but between two fires, the system obeys to the growth functions described by eqn (7) and eqn (8), only to be drawn down by the next fire. The regular reduction of shoot biomass by fire therefore causes a reduction of the long-term mean root and shoot biomasses. The level of reduction depends on the fire return interval 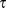 (Fig. 1C).

The maximum biomass that can on average be removed by fire is equivalent to the maximum grazing yield 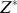 (eqn 18). In the situation where fire removes 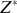, the long term mean shoot and root biomasses are 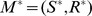 (eqn 20). Hence, despite different effects of grazing and fire on the system dynamics (stable equilibrium vs. stable limit cycles) there are no differences between grazing and fire in the long term mean.

#### Grazing and fire

**Figure 2:**
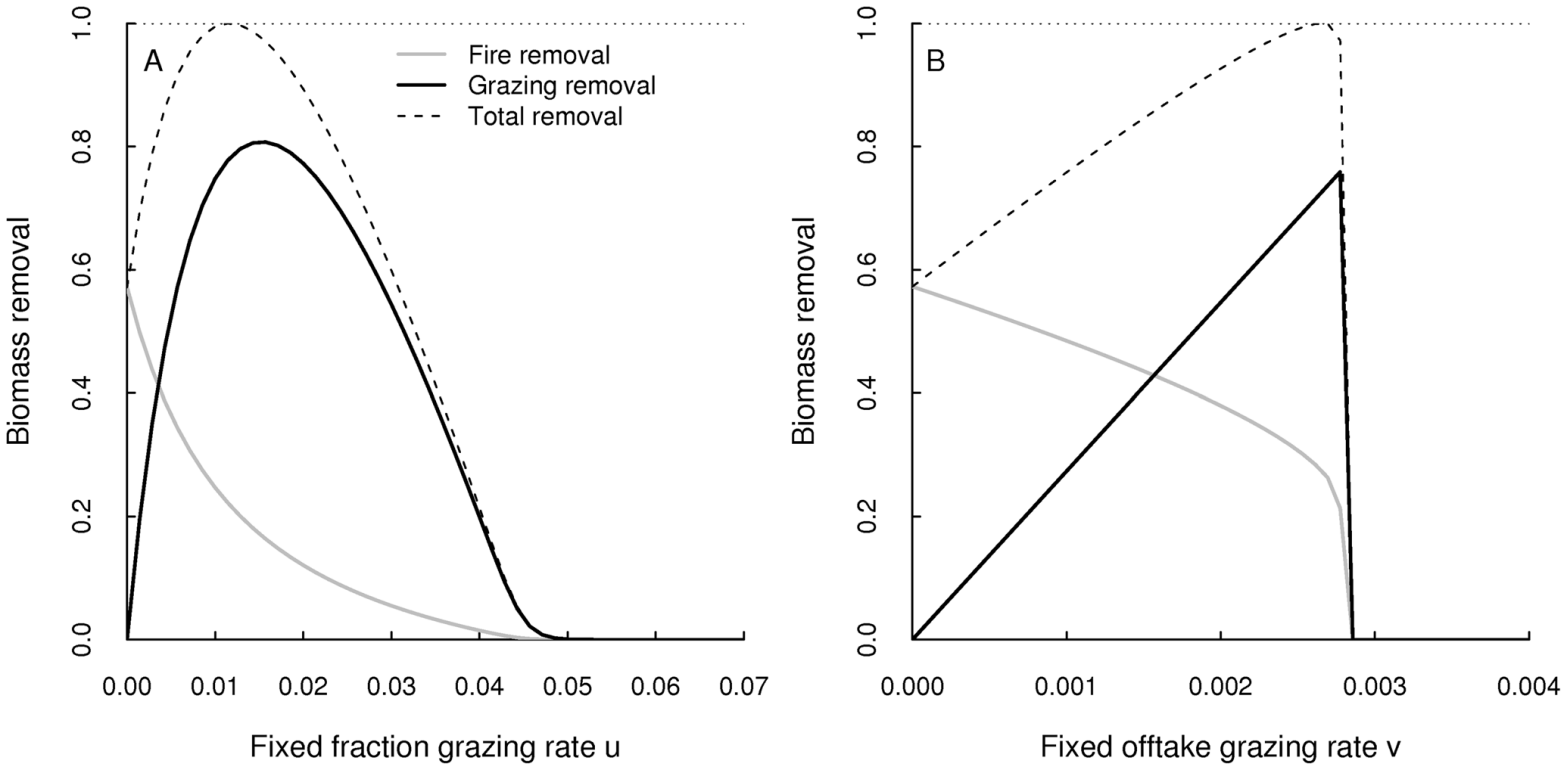
Combined grazing and fire effects in fully coupled system. Biomass removal (as a proportion of 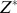) when the system is driven by fire and by fixed fraction grazing (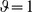, panel A) or fixed offtake grazing (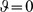, panel B). The maximum grazing yield is reduced compared to the case without fire. The maximum biomass removal of grazing and fire is 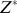. Here, 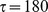.

To study the interactive effect of grazing and fire, we now analyse situations with fixed offtake grazing and fixed fraction grazing combined with fire. As would be anticipated from the previous sections, the maximum biomass removal by any combination of fire and grazing is equal to the maximum grazing yield 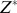. However, the maximum grazing yield is, in the selected simulation scenario, reduced to about 80% of 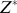 (Fig. 2). For fixed fraction grazing, the maximum grazing rate such that the system does not collapse is given by 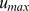 found in the situation with only grazing (compare Figs. 1A and 2A). At high grazing rates 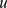, the relative effect of fire decreases as shoot biomass is, due to grazing, too low to allow significant fire effects. When the system is grazed with the fixed offtake strategy, then the maximum grazing rate 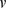 such that the system does not collapse is strongly reduced in fire driven systems compared to the fire free situation (compare Figs. 1B and 2B). Fire induces a system collapse at high grazing rates as after fire, grazing cannot be maintained. The relative impact of fire is high on the whole range of 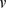.

In the case of full coupling (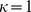), it is generally impossible to exceed the maximum biomass removal 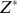, independent of the fire and/or grazing strategy. The maximum biomass removal is exclusively defined by the system characteristics (that is by 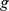 and 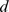) and not by the method how biomass is removed. However, fire and grazing characterise the asymptotic behaviour of the system. Fixed fraction grazing (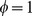) at the optimal grazing rate 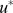 yields a resilient system as the grazing rate 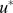 is less than the maximum grazing rate 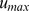. Hence, moderate disturbances such as fire do not lead to a system collapse but rather shift the equilibrium biomass slightly towards higher or lower biomass values. In contrast, when the system is grazed with fixed offtake (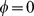) at the maximum grazing rate 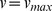, then small perturbations might induce a system collapse. In the situation of fire, shoot biomass is removed instantaneously, which imposes periodicity to the trajectory. In contrast to grazing, a fire driven system does not reach an equilibrium and the trajectory describes a stable limit cycle.

### Strong, intermediate and weak coupling

We now investigate the case 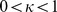. We first explore the buffering effects of roots by analysing the equilibrium root and shoot biomasses in the root-shoot phase plane at different levels of coupling 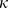 and at different grazing levels 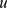. For this analysis, we use the isoclines given by
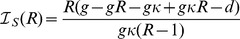
(24)

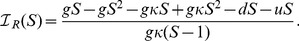
(25)


**Figure 3:**
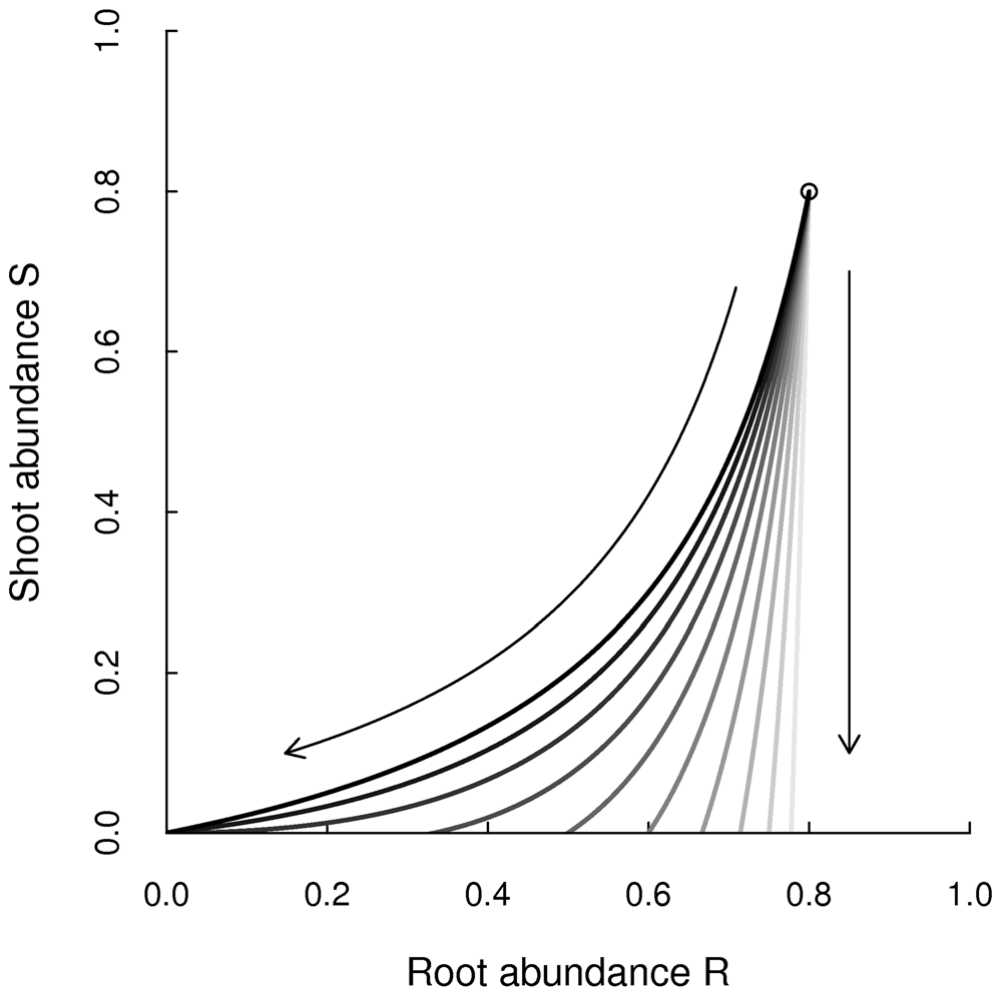
Buffering capacity of root biomass. Fixed points of the root-shoot system at different levels of grazing (the arrows indicate increasing grazing levels) and coupling (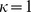 is in black, lighter grey indicates weaker coupling). The circle indicates the fixed point without grazing, which is equal for all levels of coupling.

The intersection points of the isoclines in the 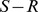 phase-plane are the fixed-points of the system. Both isoclines are functions of 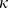, grazing only influences the isocline 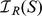. The isocline analysis shows that the root-shoot ratio is closer to a straight line between zero and the fixed-point 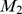 for more strongly coupled systems which indicates the higher buffering capacity of strongly coupled systems. Root biomass is lower in strongly coupled systems than it is in weakly coupled systems and therefore, shoot biomass is higher (Fig. 3). In the sections that follow we explore how the maximum biomass removal responds to different fire and grazing impacts at different values of the coupling parameter 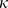.

#### Grazing

**Figure 4:**
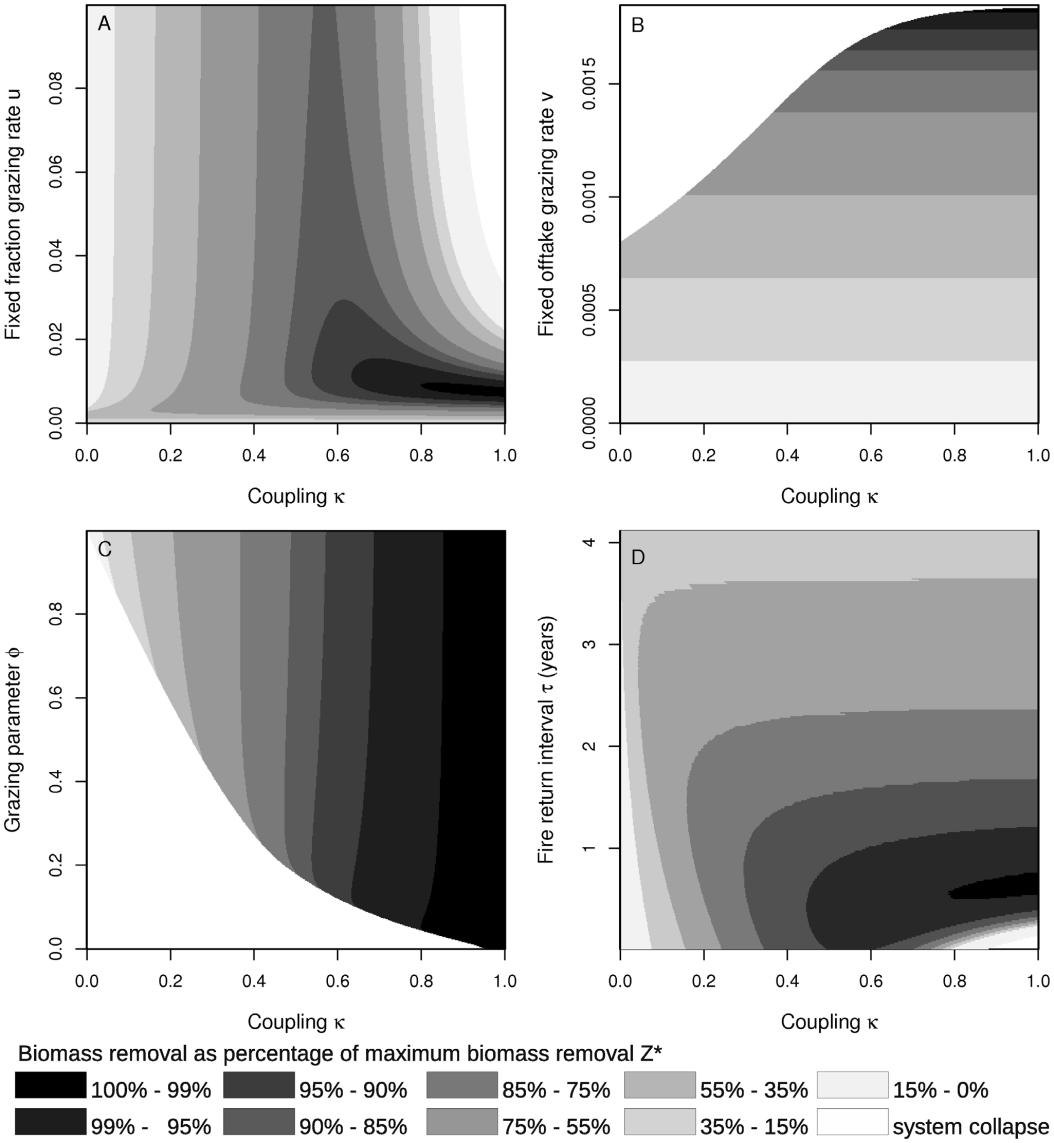
Sensitivity of biomass removal to root-shoot coupling. Biomass removal (as a percentage of 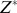) in response to grazing and fire at different levels of coupling (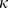). In panel A, 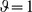 in panel B, 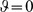 and panel C shows the grazing yield in response to coupling 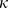 and to the mixing parameter of the grazing function 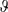. Here, the grazing function 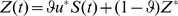 was used. Panel D depicts biomass removed by fire in response to coupling 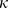 and to the fire return interval 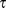.

We first consider fixed fraction grazing (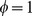). The maximum yield 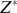 can only be obtained when shoots and roots are fully coupled that is 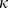 is one (Fig. 4A). However, in the case of full coupling, the maximum grazing level 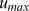 is low compared to the case where root-shoot coupling 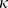 is less than one. When coupling 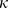 is approximately 0.6, then relatively high grazing yields (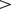
85% of 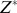) can be maintained even at grazing intensities 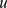 much higher than 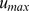. Hence, the buffering capacities of roots and the resilience of the system are maximised at intermediate levels of root-shoot coupling at the cost of a reduced maximum grazing yield.

In the situation of fixed offtake grazing (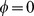), the grazing yield is, as in the case of fixed fraction grazing, maximised when 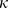 is one and it decreases as a linear function of the grazing rate 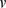 (Fig. 4B). The maximum grazing rate 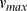 such that the system does not collapse is a non-linear function of the coupling parameter 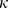.

Finally, we explore how the parameter 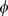, that defines the mixing of the grazing strategies influences the maximum grazing rate at different levels of coupling 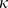. We use the grazing function 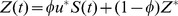 with 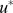 and 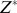 as given in eqn (19) and eqn (18). Again, the maximum grazing yield 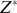 can only be obtained when shoots and roots are fully coupled (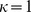). In this case, the choice of 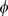 has no effect on 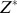 (Fig. 4C). The maximum yield decreases as 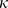 decreases and the system gets more and more unstable with respect to fixed offtake grazing (low values of 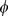). Hence, the system can be driven to collapse when coupling is weak and when the fixed offtake component of the grazing function 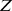 is too high.

#### Fire

Fire can remove most biomass when shoots and roots are fully coupled (Fig. 4D). However, such fully coupled systems are unstable and collapse when the fire return intervals are too short. The system is more persistent, when shoots and roots are coupled at an intermediate level. In such cases, the amount of biomass that can be removed by fire is reduced and the system is more resilient to fire.

#### Grazing and fire

We finally explore how grazing and fire interact to define the maximum biomass removal at different levels of coupling (full coupling, 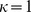; intermediate coupling, 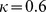; and weak coupling, 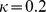). The results are consistent with the results obtained in previous sections that considered fire and grazing in isolation.

**Figure 5:**
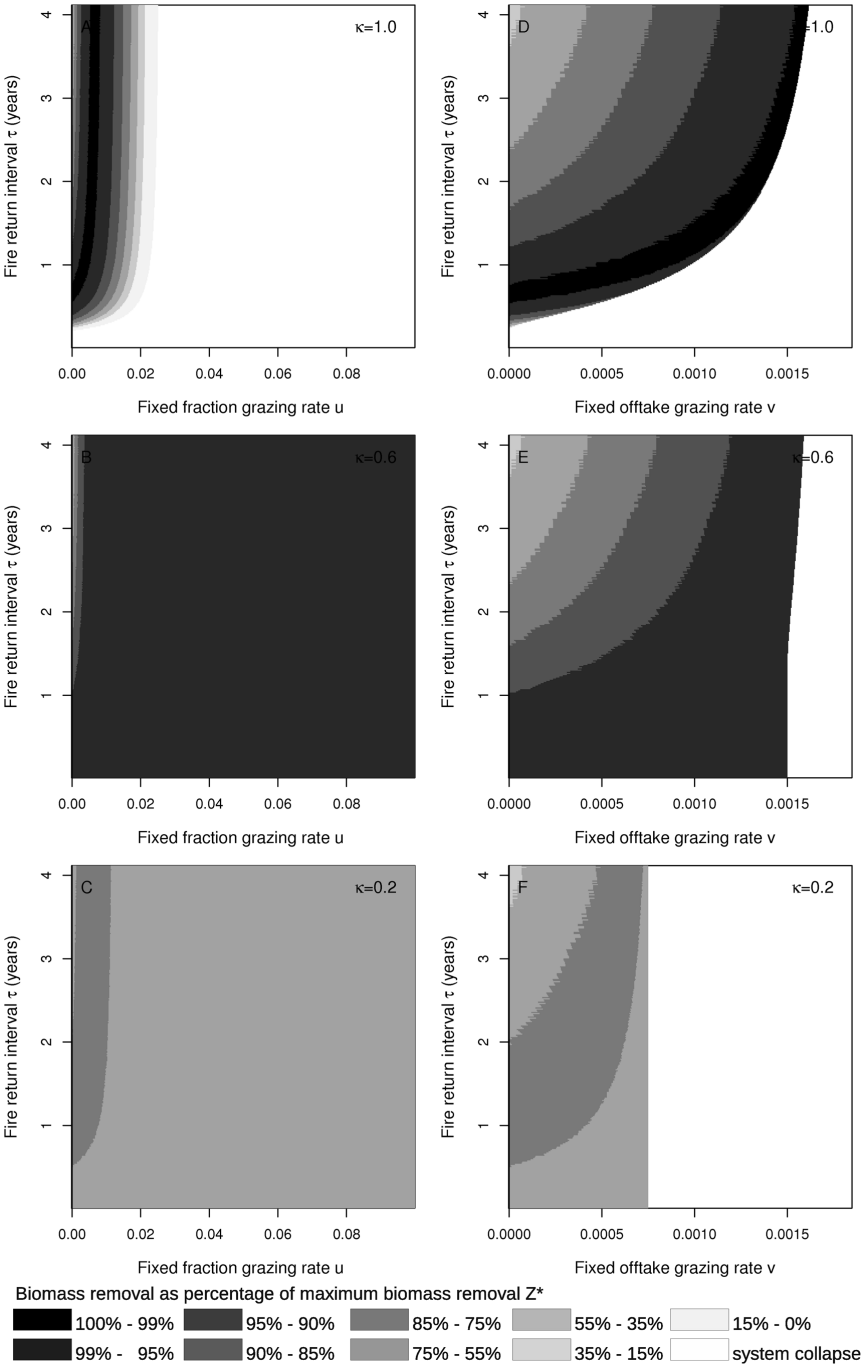
Combined grazing and fire effects in response to root-shoot coupling. Biomass removal (as a percentage of 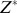) in presence of both grazing and fire at different levels of coupling (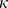). In panels A, B and C, fixed fraction grazing is applied whereas in panels D, E and F, fixed offtake grazing is applied. Coupling is strong (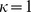) in panels A and D, intermediate (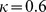) in panels B and E and weak (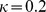) in panels C and F.

As in the situations with only fire or only grazing, biomass removal is maximised when coupling is strong, independent of the grazing strategy (Fig. 5A and 5D). However, overgrazing or short fire return intervals can easily push the system towards a collapse. When roots and shoots are coupled at an intermediate level, then the maximum biomass removal decreases whereas the parameter ranges of grazing intensities and fire return intervals that do not imply a system collapse increase (Fig. 5B and 5E). When coupling is weak, then the maximum biomass removal further decreases whereas the system is still persistent for a large range of fire and grazing regimes (Fig. 5C and 5F).

## Discussion

We explored how the coupling of aboveground and belowground biomass compartments in a grassland model influences vegetation dynamics, the grazing yield and the fire response. We found that the model system is most productive and allows the highest biomass removal when aboveground and belowground biomass compartments are fully coupled, that is when growth of roots is defined by shoots and when the growth of shoots is defined by roots. In such a fully coupled system, any mixture of grazing and fire regimes can in the long term average remove a fixed maximum biomass without inducing a system collapse. However, fully coupled systems are unstable at extreme levels of fire and grazing such that small disturbances can lead to a system collapse. Further, the case of full coupling is biologically not reasonable.

We show that the grassland system is more persistent when roots and shoots are coupled at an intermediate level, which means that both roots and shoots influence the growth of the two biomass compartments. In this situation, the feasible range of fire and grazing regimes that do not imply a system collapse increases as the level of coupling is reduced from full to intermediate. However, the cost of increasing persistence is a reduction in the maximum biomass that can be removed by herbivores and fire. The model suggests that the yield of aboveground biomass in intermediately coupled systems is 20% less than that of the fully coupled system.

The result that intermediate levels of shoot-root coupling maximise the persistence of the grassland system is plausible for several reasons. First, roots and shoots fulfil different functions and they need to exchange their products such as carbon, water and nutrients. Thus, there is empirical evidence that more than 60% of carbon fixed by photosynthesis can be allocated to roots [1] and approximately 75% of the nitrogen acquired by roots can be allocated to shoots [2]. Further, it is clear that one compartment cannot grow and survive in absence of the other compartment which is the case for weak coupling. It is also clear that growth of one compartment is not solely defined by the other compartment which is the case for full coupling. Both observations suggest intermediate levels of coupling. Second, we argue that selection should favour intermediate coupling because it increases the survival chances of individual plants in the face of disturbances such as drought, fire or herbivory [3], [26], [27]. Thus, selection should optimise a trade-off between strong coupling, which would imply highest productivity but low survivorship and weak coupling which would imply lower productivity but higher survivorship. Third, when it is assumed that roots and shoots are fully coupled then, in the model, root biomass strongly decreases at low levels of shoot biomass while shoots have high growth rates. In reality, root biomass might be more stable in the sense that it supports shoot regrowth while it remains more or less constant. In the model, this situation occurs at intermediate levels of coupling.

Despite the heuristic value of the model, it only gives a simplified representation of vegetation dynamics and the coupling of aboveground and belowground biomass. For instance, growth and decomposition are described by single parameters and without considering any ecophysiological mechanism such as photosynthesis and respiration [28]. Coupling is only described by parameters describing how vegetation growth is co-limited by different biomass compartments and how one biomass compartment supports regrowth after disturbances. More complex interactions between aboveground and belowground biomass [29], [30] or stoichometric constraints for the C:N:P ratio that influence for instance palatability of grasses and thereby trophic interactions [31] are ignored. More complex models that include more plant compartments, explicit resource dynamics, root herbivores, complex allocation patterns or leaf physiology have been developed [7], [8], [12], [30], [32]–[36]. These models allow to establish a tighter link between data and models, allowing quantitative testing of the ideas developed here. However, one problem of more complex models is that it is more difficult to dissect out the influence of single mechanisms as complex simulation results are influenced by many processes and interactions.

Heuristic models such as the model presented here are generally difficult to parametrise and validate. One reason is that the parameters used in the model describe the aggregated outcome of several underlying ecological processes. Nonetheless, the model could be parametrised and validated by conducting field experiments that measure the transfer rates of metabolites between aboveground and belowground organs. An alternative approach is to parametrise the model indirectly [37], [38] by fitting the model to data that describe how the abundances of aboveground and belowground compartments of a grassland ecosystem respond to different fire regimes and herbivory [39]. Such a parametrised model would allow us to test the hypothesis that intermediate coupling optimises the cost-benefit relation of persistence and productivity.

Despite the simplicity, the model provides valuable insights to grassland dynamics and to the response of grasslands to disturbances. Such insights differ from those of single compartment models which form the foundation of theoretical and applied ecology [6].
